# Computational Modeling of the Trauma Injury

**DOI:** 10.1155/2015/679525

**Published:** 2015-10-05

**Authors:** Feng Zhu, Clifford C. Chou, Libo Cao

**Affiliations:** ^1^Department of Mechanical Engineering, Embry-Riddle Aeronautical University, Daytona Beach, FL, USA; ^2^Bioengineering Center, Wayne State University, Detroit, MI, USA; ^3^State Key Laboratory of Advanced Design and Manufacturing for Vehicle Body, Hunan University, Changsha, Hunan 410082, China

Traumatic biomechanics of the human body under various loading conditions, such as car crash, falling, contact sports, and military environments, has been studied for decades. The goal of research in this area is to advance knowledge pertaining to human injury causation and develop affordable and effective countermeasures for protection towards injury and fatality reduction. From an ethical point of view, it is extremely difficult to propose a well-controlled human subject study aiming at understanding the injury mechanisms and establishing the associated tolerance values. For this reason, many numerical simulation techniques, such as finite element, boundary element, and meshless methods, have been used to study the human body response in an attempt to obtain in-depth insights into injury biomechanics, thus minimizing the need for testing human subject in this important research field.

This special issue serves as a brief update to the current status of and advances in using commercially available numerical tools in the study of human body responses under different impact situations and development of injury prevention measures to protect their respective human body regions.

The paper by X. Song et al. analyzes the biomechanical effects of sinuses in the skull on the facial impact response. Two finite element head models with and without sinuses are built. The results show that if the sinuses are far away from the impact location, its effect could be neglected. Another paper by the same group investigates the influence of gyri and sulci. The responses of two head models, with and without gyri and sulci under impact loading conditions, are compared. The model predictions indicate considerable changes of stress and strain between two cases, thus pointing out the necessity to include these anatomic features in the modeling. S. Cui et al. study how the variation of pediatric brain tissue properties can affect the intracranial response of a 6-year-old child in the impact accidents. The bulk modulus and viscoelastic parameters are changed in a large wider range and the simulated responses show an opposite trend between the shear stress and shear strain distributions. More investigation is needed. Y. Hua et al. develop a computational model to study the role of network of blood vessels in the brain under air blast loading. Voronoi tessellations are implemented to represent the network of brain. The results demonstrate an evident increase of strains when the blood vessels are included. But they have limited effect on the intracranial pressure. D. Li et al. compare the responses of an isolated head finite element model and a full-body model with head under the same impact loading conditions. Based on the results, they conclude that the isolated head model may not equivalently reflect the strain levels below the 10% compared to the whole human body model. J. Lei et al. use detailed finite element models to evaluate the mechanism of pelvis ring fracture. It has been found that the pelvic ring integrity is the prerequisite of the pelvic stability and should be in a stable condition when the complex fracture is treated. Y. Fan et al. apply numerical simulations to evaluate the performance of fixation systems in the most frequent T-shaped acetabular fracture and an optimal design of fixture is achieved. M. Sridevi et al. predict healing period of a tibia fracture in humans across limb using first-order mathematical model. The computational results confirm the X-ray diagnostic data.

The papers included in this special issue present some recent advances of computational modeling tools in the traumatic injury related biomechanical and clinical studies. With the rapid development of modern computers and computational methods, novel modeling/simulation techniques and new applications of numerical models are emerging. We hope that the readers will find articles in this special issue interesting and stimulating enough to encourage them in directing efforts toward fostering further research on computational methods for the trauma injury in the near future.

*Feng Zhu* *Feng Zhu* *Clifford C. Chou* *Clifford C. Chou* *Libo Cao* *Libo Cao*

